# Multi-view deep fusion for forensic dental age estimation in tomographic imaging

**DOI:** 10.1007/s00414-026-03749-6

**Published:** 2026-03-10

**Authors:** Larissa Ferreira Rodrigues Moreira, Rodrigo Moreira, Leonardo Gabriel Ferreira Rodrigues

**Affiliations:** 1 Institute of Exact and Technological Sciences, Federal University of Viçosa (UFV), Rio Paranaíba, Minas Gerais Brazil; 2 School of Computer Science, Federal University of Uberlândia (UFU), Uberlândia, Minas Gerais Brazil

**Keywords:** Forensic odontology, Dental age estimation, Deep learning, Multi-view fusion, Cone beam computed tomography

## Abstract

Accurate Dental Age Estimation (DAE) is fundamental for forensic identification, particularly regarding legal age thresholds. This study is intended for forensic decision support using CBCT scans that are already available or acquired for justified clinical or medico-legal purposes, rather than advocating CBCT acquisition solely for age estimation. While Cone Beam Computed Tomography (CBCT) provides high-resolution volumetric data, current deep learning methods often rely on single-planar analysis, neglecting the anisotropic changes in the dental pulp cavity. We propose a multimodal deep fusion approach that integrates orthogonal cone-beam computed tomography (CBCT) views (coronal and sagittal) with clinical metadata to support forensic age assessment. Our dual-stream architecture, based on EfficientNet-V2, extracts visual features from maxillary central incisors and fuses them with biological sex and an automated Pulp-to-Tooth Ratio (PTR) index. Evaluated on the IPCTI dataset, the framework demonstrated a specialized performance profile: while the single-view model achieved superior global stability (MAE 5.49 years), the proposed multi-view fusion established a new state-of-the-art for the young adult demographic (18–32 years), reducing the MAE by up to 31.3% in the youngest cohort. Grad-CAM interpretability confirmed that the network targets biologically relevant markers, specifically the pulp chamber and cervical root canal. This approach advances automated DAE by providing a reproducible research prototype and benchmark evidence for CBCT-based DAE in forensic medicine settings.

## Introduction

Forensic Dental Age Estimation (DAE) is a foundational task in human identification, supporting disaster victim identification, missing-person investigations, and medico-legal decision-making when documentary records are absent or disputed [1–3]. In adults, teeth remain particularly informative because age-related physiological changes, most notably secondary dentin deposition, progressively reduce the pulp space, creating measurable anatomical signatures [2, 4].

The increasing use of Cone Beam Computed Tomography (CBCT) in dental care has also expanded the availability of three-dimensional tomographic imaging, enabling the quantitative assessment of internal tooth structures beyond what is feasible in standard radiographs [5]. Consequently, CBCT-based DAE has become a relevant direction, particularly when CBCT data are already available from justified clinical or medico-legal imaging, with the potential to deliver more objective and reproducible assessments than conventional approaches [3, 6].

This study targeted forensic dental age estimation from existing or newly acquired CBCT data for justified clinical or medico-legal reasons. We do not propose the use of CBCT as a screening tool for age assessment. The method is applicable in two common settings: age estimation in living persons when age is disputed and CBCT is already available from dental care or clinically indicated imaging, and age estimation in unknown deceased persons when tomographic imaging is part of postmortem identification workflows. These settings differ in radiation protection requirements, but they share the same anatomical substrate used by our model, the secondary dentin-related reduction of the pulp space.

Beyond dentistry, the broader landscape of medical image analysis has been transformed by the rapid evolution of Deep Learning (DL). Convolutional Neural Networks (CNNs) and transformer-based architectures have achieved state-of-the-art performance in complex diagnostic tasks across oncology, radiology, and cardiology, often matching or exceeding human-level expertise in pattern recognition. Recent advances have moved toward multi-view and multimodal fusion strategies, where models simultaneously process volumetric and clinical metadata to improve diagnostic sensitivity [7–10]. These general medical advancements provide a robust technological foundation for forensic odontology, suggesting that specialized neural architectures can effectively model the subtle and nonlinear morphological changes required for high-precision age estimation.

Despite the potential of current CBCT-based adult DAE methods, a discrepancy remains between their reported accuracy and the evidential reliability required in forensic contexts. Many methodologies extract data from a single plane or a limited representation of the tooth, even though age-related changes manifest differently in orthogonal views [2, 11]. This can result in unreliable predictions when anatomical variability, slice selection, or image artifacts obscure critical regions in a single view. The literature is often divided into two largely disconnected categories: (i) geometric approaches focused on pulp-related indices, such as the Pulp-to-Tooth Ratio (PTR), which are biologically meaningful but sensitive to measurement noise and limited in representational capacity, and (ii) end-to-end DL regressors, which can capture complex patterns but frequently lack interpretable connections to established forensic indicators and may generalize inconsistently across age cohorts [3, 5, 12].

Furthermore, direct progress is challenging to quantify because studies often employ different validation protocols, and performance can vary significantly when evaluated under repeated cross-validation using the same benchmark dataset. Collectively, these limitations present an ongoing challenge in developing a CBCT-based adult DAE method that effectively leverages complementary multiplanar evidence, remains grounded in interpretable biological markers, and is validated under a protocol that facilitates fair and direct comparison.

To address this issue, we developed and validated a Multi-View Deep Fusion approach for forensic DAE, which integrates coronal and sagittal CBCT slices with clinical metadata (sex and automated PTR) to enhance estimation accuracy across the adult lifespan. Our approach explicitly combines deep visual features with clinical metadata, including biological sex and the PTR, ensuring that the model is grounded in recognized biological patterns of secondary dentin deposition. Furthermore, our proposed multiview fusion approach mitigates the limitations of significant error spikes, particularly in older cohorts, in which pulp narrowing is subtle. Although our method is implemented with modern deep learning components, the manuscript is written with a forensic audience in mind. Therefore, we focused on the practical interpretation of CBCT-based DAE, including cohort-specific error behavior and reliability diagnostics relevant to legal thresholds, and we kept technical details only to the extent needed for reproducibility. The main contributions of this study are as follows:

- A dual-stream deep fusion architecture for high-precision age estimation in young adults.
- An automated pipeline was implemented for PTR extraction from expert-validated segmentation masks to guide the learning process.
- A performance benchmark was established using a repeated 5-fold cross-validation protocol on the Incisor Pulp Chamber Tomographic Images (IPCTI) dataset to ensure statistical robustness.
- A comparative analysis suggested that while multi-view synchronization is essential for complex pulp geometries, single-view architectures remain more resilient as a generalist approach across the full adult lifespan.
- An explainability audit using visual saliency maps to confirm that the model’s predictions are based on relevant forensic dental structures

This study is a feasibility and benchmarking investigation of a public dataset; the proposed model is not intended for direct forensic deployment and requires further external validation before practical use.

The remainder of this paper is organized as follows. Section “Related work” reviews the related literature. Section “Material and methods” describes the material and methods. Section “Results” presents the experimental results and Section “Discussion” discusses the findings. Finally, Section “Conclusion” concludes the paper and outlines directions for future work.

## Related work

Recent advancements in CBCT-based DAE generally follow two trajectories: segmentation-driven volumetric analysis and feature-engineered machine learning [2–4]. Du et al. [13] utilized deep-learning-based 3D segmentation to quantify pulp chamber volume, applying logarithmic regression to model age across diverse populations and achieving a within-population Mean Absolute Error (MAE) of 8.43 years. Similarly, [14] targeted multiradicular first molars, employing a U-Net to reconstruct 3D pulp cavities from sagittal slices. Their volumetric biomarker approach yielded an MAE of 6.72 years, although biological sex was treated only as a post-hoc covariate rather than an integrated predictor.

Alternative approaches rely on manual or automated morphometric descriptors. Saric et al. [15] extracted tabular predictors from craniofacial and dentoalveolar structures such as alveolar bone levels and maxillary sinus volumes achieving an MAE of 6.02 years via Random Forest regressors. Senol et al. [16] further integrated explainability into this process, utilizing SHAP (SHapley Additive exPlanations) to analyze the impact of quantitative measurements on SVM-based age inference and sex classification.

More recent studies have transitioned to end-to-end DL. Pishghadam et al. [17] proposed a multi-task CNN framework with attention mechanisms to jointly predict age and sex from 2D slices. Although they reported a high correlation, the model relied on single-stream 2D features rather than volumetric or multiplanar integration. Pacelli et al. [18] addressed this by developing a three-stage pipeline (YOLO-based detection, Attention U-Net segmentation, and feature fusion) using the IPCTI dataset. Their multiview approach, focusing on the upper central incisors, achieved an MAE of 4.9 years by fusing morphometric descriptors extracted from segmented masks.

Despite these efforts, current models often treat multiplanar data as independent inputs or rely heavily on the precision of intermediate segmentation masks, which are prone to artifacts. Furthermore, biological sex is frequently modeled as an auxiliary output task rather than a fundamental prior in the regression space.

Table 1 provides a comparative synthesis of the proposed method with representative state-of-the-art studies in CBCT-based dental age estimation. The column “CBCT Based” identifies frameworks utilizing volumetric data, which provides superior spatial resolution for pulp cavity analysis; “Dual-Stream DL” specifies the use of parallel processing pipelines that learn features from multiple inputs simultaneously; “Ortho-Sync Fusion” denotes the integration of synchronized orthogonal planes (coronal and sagittal), allowing the model to capture anisotropic dentin deposition; “PTR-Clinical Fusion” refers to the late-stage integration of biological sex and the automated PTR index into the regression head; and “High Precision” indicates a MAE below 4.0 years, a critical threshold for forensic reliability in the young-adult demographic.

**Table 1:** Comparison with representative studies and with approaches that are directly comparable under the IPCTI benchmark setting

Study	CBCT Based	Dual-Stream DL	Ortho-Sync Fusion	PTR-Clinical Fusion	High Precision (MAE < 4.0)
Du et al. [13]	⬤	○	○	○	○
Saric et al. [15]	⬤	○	○	○	○
Senol et al. [16]	⬤	○	○	○	○
Song et al. [14]	⬤	○	○	○	○
Pishghadam et al. [17]	⬤	○	○	○	⬤
Pacelli et al. [18]	⬤	○	○	○	○
Our Proposal	⬤	⬤	⬤	⬤	⬤

Table 1 focuses on the closest comparable setting, while these modalities are treated as complementary. Prior studies have estimated age using 3D CBCT-derived pulp/tooth volumes (or volumetric ratios), and radiation-free MRI feasibility studies have also been conducted to visualize pulp changes. However, these studies were typically evaluated using different datasets and protocols, limiting direct numerical comparability with the IPCTI benchmark used here.

### Contribution positioning

Compared with the previously mentioned approaches, our method introduces a dual-stream EfficientNet architecture that learns synchronized orthogonal representations from the coronal and sagittal planes. Unlike segmentation-only methods, our model extracts deep latent features that capture non-linear secondary dentin deposition patterns that are often missed by handcrafted volumetric proxies. While [18] utilized multi-view detection, our approach employed late-stage clinical fusion, explicitly integrating an automated PTR index and biological sex into the feature space. This synergy allows the model to emulate the multiplanar assessment performed by forensic experts, resulting in a significant performance improvement.

## Material and methods

### Study design and pipeline overview

**Fig. 1:**
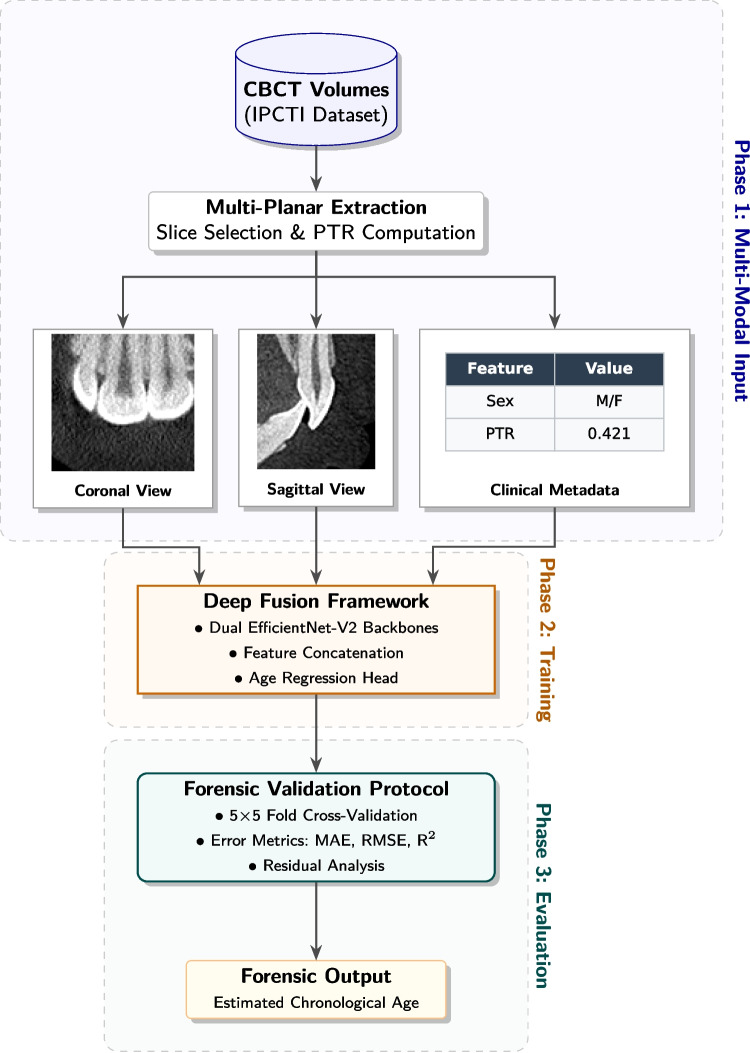
Overview of the proposed method

We propose a multimodal deep fusion method for forensic dental age estimation from CBCT, modeling anatomy through coronal and sagittal planes (Fig. 1).

Starting from an input CBCT volume, the pipeline extracts two orthogonal views centered on the upper central incisors, yielding a coronal slice and a sagittal slice, and computes the PTR along with clinical metadata (Phase1). These three information sources are then processed by a deep fusion architecture (Phase2) consisting of dual EfficientNet-V2 backbones for view-specific feature learning and a lightweight metadata encoder; their embeddings are fused via feature concatenation and mapped to an age regression head. Finally, the model was assessed using a forensic validation protocol (Phase3) based on repeated 5-fold cross-validation and agreement-oriented residual analysis, producing an estimated chronological age and reliability diagnostics.

Our approach integrates high-capacity representation learning with forensic interpretability by incorporating an automated PTR, a biologically grounded index commonly used in adult DAE, and clinical metadata (biological sex) directly into the regression space. Furthermore, the method was designed for benchmark-grade evaluation, facilitating direct comparison under a repeated cross-validation protocol consistent with the IPCTI guidelines.

Algorithm 1 summarizes the orthogonal view extraction and late fusion strategies. We encoded coronal and sagittal slices with view-specific backbones and fused the resulting embeddings only after independent feature learning, which preserved complementary evidence and reduced sensitivity to view-dependent artifacts.

**Algorithm 1:**
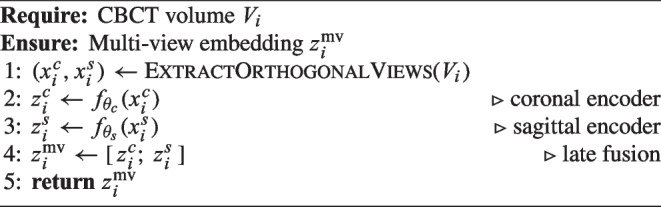
Multi-view feature extraction and late fusion.

Formally, the view encoders produce

1 $$\begin{aligned} z_i^{c} = f_{\theta _c}(x_i^{c}), \qquad z_i^{s} = f_{\theta _s}(x_i^{s}), \end{aligned}$$

Late fusion is implemented by concatenation as follows:

2 $$\begin{aligned} z_i^{\text {mv}} = [\, z_i^{c};\, z_i^{s} \,]. \end{aligned}$$

To incorporate forensic priors, we appended the clinical metadata vector $m_i = [\text {PTR}_i; \text {Sex}_i]$ before the regression head:

3 $$\begin{aligned} Z_{total} = [\, z_i^{\text {mv}};\, m_i \,]. \end{aligned}$$

The resulting representation is mapped to the final age estimate $\hat{y}_i$ by the Multi-Layer Perceptron (MLP) regressor.

### Model architecture

**Fig. 2:**
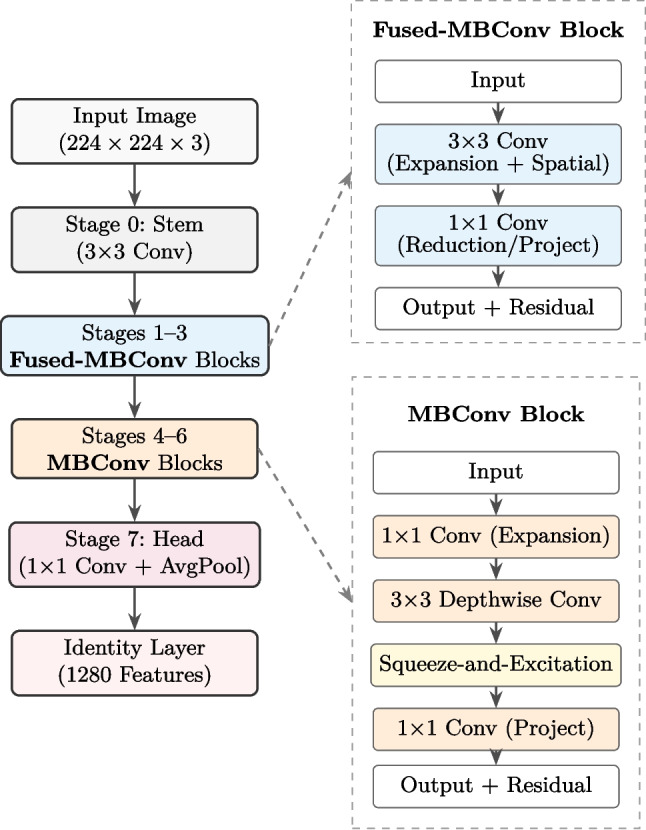
Detailed architecture of the EfficientNet-V2-S backbone utilized for feature extraction. The diagram highlights the macro-stages (left) and the internal structure of Fused-MBConv and MBConv blocks (right), which optimize feature capture for secondary dentin analysis

Visual feature extraction was performed using the EfficientNet-V2 Small (V2-S) architecture, which represents an evolution in CNN optimized for training efficiency and parameter scaling [19]. Unlike its predecessor, the V2-S variant utilizes Fused-MBConv blocks in the early layers, which replace the depthwise convolutions with standard 3$\times$3 convolutions to improve the computational throughput and feature representation in medical imaging tasks (Fig. 2).

In the initial stages (Stages 1–3), the network utilizes Fused-MBConv blocks, which merge the expansion and depthwise convolutions into a single 3$\times$3 operator to improve feature richness and computational efficiency in the early layers. As the signal propagates to deeper stages (stages 4–6), the model transitions to standard MBConv blocks. These blocks incorporate Squeeze-and-Excitation (SE) modules, which adaptively recalibrate channel-wise features, allowing the network to prioritize the subtle textural boundaries of the dental pulp cavity over the surrounding noise. The process culminates in a global average pooling stage that produces a 1280-dimensional embedding that serves as a refined visual signature for subsequent age regression.

**Fig. 3:**
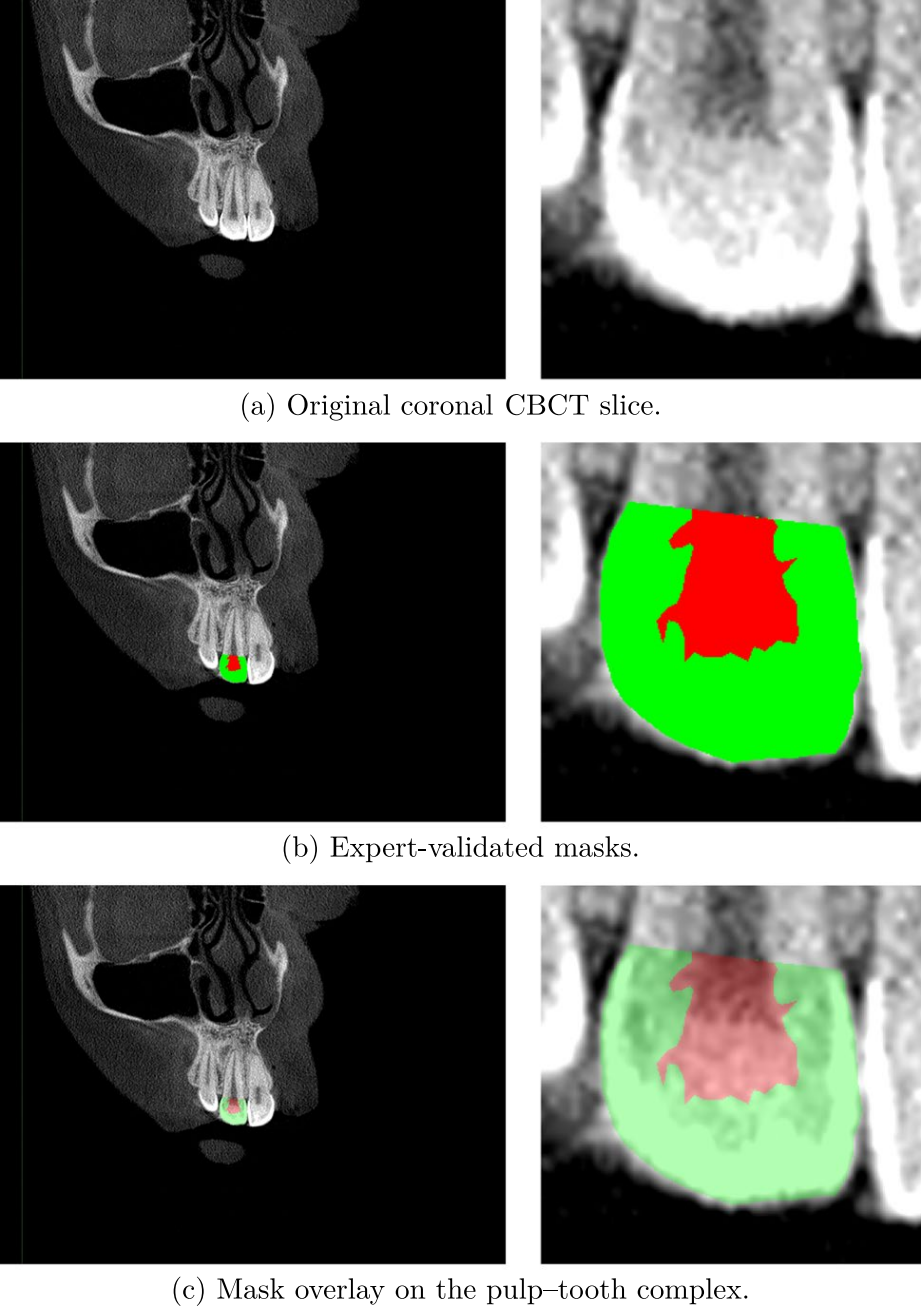
Example of expert-validated annotations provided with the IPCTI dataset. Left: full field-of-view. Right: magnified view of the same region

### Dataset

We used the Incisor Pulp Chamber Tomographic Images (IPCTI) dataset^1^, a public CBCT-based benchmark designed for forensic dental age estimation in adults. The dataset comprises scans from $N{=}662$ individuals (452 female, 210 male) aged 18–60 years, collected in Juiz de Fora, Minas Gerais, Brazil, under a standardized acquisition and alignment procedure. For each subject, the dataset provided images of the two upper central incisors (teeth 11 and 21) in both the coronal and sagittal planes, totaling four images per subject and 2,648 images overall [18, 20].

The segmentation masks (tooth and pulp) were not produced by the authors of this study but were provided by the public IPCTI release. The dataset documentation describes these masks as expert-produced and includes qualitative examples; however, it does not report the annotators’ professional profiles (e.g., dentist, dentomaxillofacial radiologist, radiographer, or forensic practitioner) or their years of experience. Therefore, we treat the masks as third-party reference annotations released by IPCTI, and we do not make claims beyond what is documented in the dataset description.

Table 2 summarizes the demographic distribution of the study population stratified by age cohort and biological sex. The dataset spans the full adult range (18–60 years) and exhibits a realistic imbalance, with fewer individuals in older cohorts. This distribution is particularly relevant for forensic evaluation, as it can influence error profiles across age ranges and motivates reporting stratified results. This imbalance increases the uncertainty of cohort-specific estimates, particularly in sparsely represented age ranges; therefore, the results of these cohorts should be interpreted with caution. Accordingly, we treated the findings as benchmarked feasibility evidence of IPCTI rather than definitive population-level performance.

**Table 2:** Demographic profile of the IPCTI by age group and sex

Age Group	Male	Female	Total	Percentage
18-22	24	74	98	14.80%
23-27	32	72	104	15.71%
28-32	27	51	78	11.78%
33-37	33	55	88	13.29%
38-42	27	60	87	13.14%
43-47	27	48	75	11.33%
48-52	17	44	61	9.21%
53-60	23	48	71	10.73%
Total	**210**	**452**	**662**	**100.00%**

Figure 3 exemplifies the annotation resources distributed in the IPCTI dataset. It shows a representative coronal CBCT slice together with the corresponding expert-validated ground-truth masks and their visual overlays, including a zoomed view to emphasize boundary placement within the pulp-tooth complex. The superimposed masks highlight the delineation of clinically relevant structures, particularly the pulp region relative to the surrounding tooth tissues, which is critical for age estimation based on the pulp alone. These annotations provide a standardized reference for reproducible evaluation and facilitate downstream morphological measurements, such as the computation of the PTR, across the full cohort.

### Evaluation protocol and metrics

To ensure direct comparability with prior IPCTI-based benchmarks and to avoid subject-level leakage, we followed the official stratified 5$\times$5 cross-validation protocol proposed by [18]. In this setup, all images from the same patient are strictly assigned to the same fold. The five cross-validation sets differ only by the random seed while preserving the overall age distribution across folds.

The predictive performance was summarized using MAE and RMSE (in years) and $R^2$, and complemented by residual analysis across age cohorts to reveal systematic over- or under-estimation patterns relevant to forensic interpretation.

### Implementation details

The experimental setup was implemented using Python 3.8 and PyTorch 2.4 and executed on the Fabric testbed [21], utilizing a high-performance virtual environment equipped with 32 vCPUs, 32 GB RAM, and an NVIDIA Quadro RTX 6000 GPU (CUDA 12.2). The EfficientNet-V2 Small (V2-S) backbone was initialized with pretrained weights from ImageNet [22] to leverage the transfer learning. Simultaneously, clinical metadata (PTR and biological sex) were processed using a dedicated MLP comprising a linear layer, Rectified Linear Unit (ReLU) activation, and a dropout layer for regularization. Visual features and metadata embeddings were integrated via late fusion to create a high-dimensional representation for the final regression head. All evaluations followed the subject-level $5\times 5$ repeated cross-validation protocol described in the previous subsection.

## Results

The training was conducted for 30 epochs with a batch size of 16 using the Adam optimizer ($LR=10^{-4}$). To align the optimization objective with the forensic requirements, L1 Loss (MAE) was employed as the criterion. The input images were resized to $224 \times 224$ pixels and normalized following the standard ImageNet distribution.

### Performance metrics and regression analysis

The predictive accuracy was evaluated using the IPCTI dataset ($N=662$ subjects). Following the $5\times 5$ repeated cross-validation protocol, 3,310 age estimations were generated. Ground truth annotations followed the IPCTI-provided annotations by [18], ensuring high-fidelity PTR extraction.

**Fig. 4:**
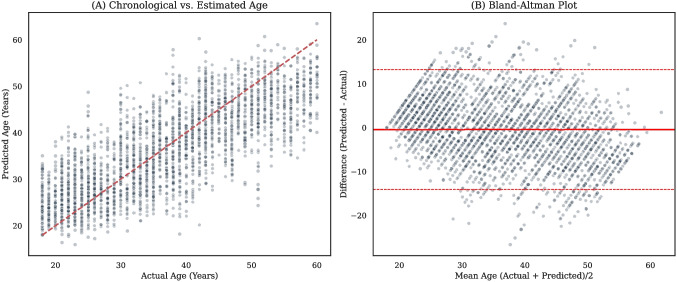
Global predictive performance analysis of the multi-view deep fusion model

The global regression analysis for the single-view model yielded a MAE of $5.49 \pm 4.32$ years and a coefficient of determination ($R^{2}$) of 0.641 (Fig. 4). The Multi-View architecture achieved a comparable global MAE of $5.56 \pm 4.51$ years. Bland–Altman analysis revealed a mean bias near zero, with limits of agreement (95% LoA) ranging from approximately $-14.1$ to $+13.5$ years. The distribution of residuals showed a characteristic “attraction to the mean” effect, with a progressive transition from slight overestimation in younger subjects to underestimation in the senior cohort, correlating with the biological variability inherent to dental mineralization.

Detailed diagnostic metrics, including MAE, Root Mean Square Error (RMSE), and $R^{2}$ for both proposed architectures across all age cohorts, are summarized in Table 3. The proximity between the MAE and RMSE values in the younger cohorts (18–32 years) indicates a low prevalence of large prediction outliers, reinforcing the reliability of multi-view fusion for these groups. Conversely, the increased RMSE in the senior cohorts (53–60 years) reflects the higher biological variability and the impact of tomographic artifacts on estimation stability.

**Table 3:** Detailed diagnostic metrics for the proposed models

	Single-View (Ours)		Multi-View (Ours)	
Age Group	MAE (yrs)	RMSE (yrs)	MAE (yrs)	RMSE (yrs)
18–22	5.55	6.84	4.91	6.16
23–27	4.14	5.72	4.12	5.51
28–32	4.87	6.17	4.46	5.90
33–37	4.80	5.95	5.13	6.23
38–42	5.50	7.05	5.30	6.78
43–47	5.25	6.57	5.38	6.80
48–52	5.77	7.38	6.34	8.05
53–60	8.90	10.16	10.18	11.60
Global Metrics	**5.49**	**6.98**	**5.56**	**7.16**
$R^{2}$	**0.641**		**0.622**	

### Age-group stratification and model comparison

The comparative performances of the IPCTI benchmark baseline reported by [18] and our proposed architectures are presented in Table 4.

**Table 4:** Comparative performance (MAE ± SD) for dental age estimation. The bold values indicate the best performance for each cohort. SD values reflect population-based predictive uncertainty

Age Group	Count (*N*)	Baseline [18]	Single-View (Ours)	Multi-View (Ours)
18–22	490	7.15	5.55 ± 4.00	**4.91 ± 3.73**
23–27	520	5.58	4.14 ± 3.95	**4.12 ± 3.66**
28–32	390	4.81	4.87 ± 3.80	**4.46 ± 3.86**
33–37	440	**4.64**	4.80 ± 3.51	5.13 ± 3.54
38–42	435	**5.01**	5.50 ± 4.42	5.30 ± 4.23
43–47	375	**4.61**	5.25 ± 3.95	5.38 ± 4.16
48–52	305	6.16	**5.77 ± 4.61**	6.34 ± 4.96
53–60	355	9.54	**8.90 ± 4.90**	10.18 ± 5.57
Global MAE	**3310**	5.89	**5.49 ± 4.32**	5.56 ± 4.51

The multi-view model established a new benchmark for the young adult demographic (18–32 years), significantly reducing the error in the 18–22 group from 7.15 to 4.91 years. This represents a substantial advancement over geometric feature baselines. Conversely, the Single-View model exhibited superior global stability (MAE 5.49) and better performance in senior cohorts (48–60 years).

To quantify these advancements further, an improvement analysis was conducted (Table 5). The most significant gain was observed in the 18–22 cohort, where the proposed framework achieved a 31.3% error reduction ($\Delta = -2.24$ years).

**Table 5:** Improvement analysis. Negative values ($\Delta$) indicate an improvement in MAE

Age Group	Baseline [18]	Best Proposed	Model	$\Delta$ MAE (yrs)	Gain (%)
18–22	7.15	4.91	Multi-View	${-2.24}$	**31.3%**
23–27	5.58	4.12	Multi-View	$-1.46$	26.2%
28–32	4.81	4.46	Multi-View	$-0.35$	7.3%
48–52	6.16	5.77	Single-View	$-0.39$	6.3%
53–60	9.54	8.90	Single-View	$-0.64$	6.7%
Global	**5.89**	**5.49**	**Single-View**	${-0.40}$	**6.8%**

### Residual analysis and interpretability

**Fig. 5:**
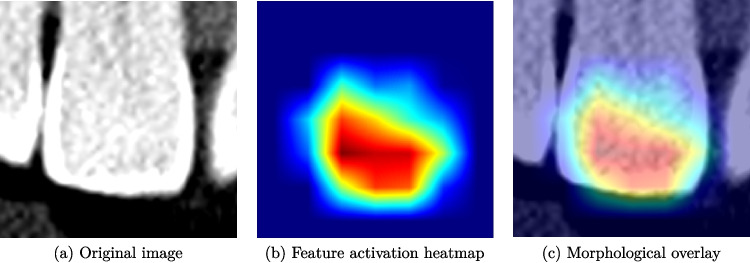
Visual interpretability analysis using Grad-CAM

**Fig. 6:**
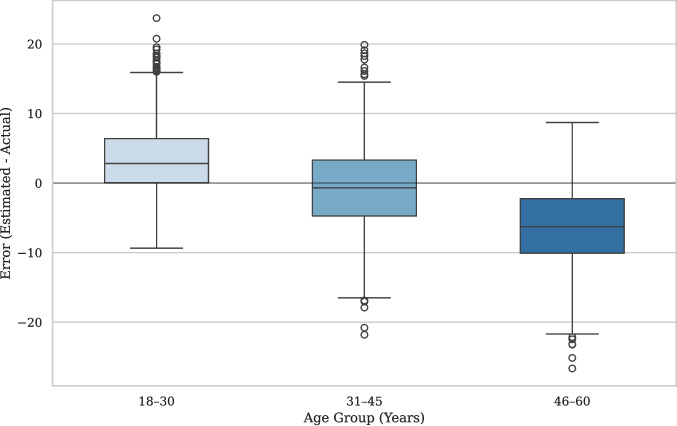
Predictions residual by age group

Visual interpretability via Gradient-weighted Class Activation Mapping (Grad-CAM) [23] confirmed that the models successfully identified the biologically relevant features. Activation heatmaps (Fig. 5) consistently focused on the coronal pulp chamber and the cervical root canal, areas where secondary dentin deposition is most pronounced. Residual analysis (Fig. 6) indicated that while dispersion increases with age due to biological variability, the proposed DL approach maintains a tighter error distribution compared to previous methods.

## Discussion

The high precision attained by the multi-view model in the young adult demographic (18–32 years) underscores the critical role of multiplanar feature fusion in forensic DAE. Specifically, the reduction of the MAE in the 18–22 group from 7.15 years (baseline) to 4.91 years represents a landmark improvement of 31.3% (Table 5). In early adulthood, secondary dentin deposition is a highly dynamic and often asymmetrical process. By integrating synchronized coronal and sagittal slices, our dual-stream EfficientNet-V2-S architecture effectively captured the anisotropic nature of dental-pulp cavity reduction. This volumetric perspective allows the network to detect subtle morphological markers, such as the narrowing of the pulp horns and initial floor mineralization, which are frequently underestimated in traditional geometric or single-planar projections [18]. Such precision is important for legal proceedings, where a 2.24-year error reduction can significantly impact the determination of the legal majority (18- or 21-year thresholds).

The robustness of the proposed method is demonstrated by the detailed diagnostic metrics in Table 3. The close alignment between the MAE and RMSE values in the younger cohorts (18–32 years) is a noteworthy finding for forensic applications, as it indicates a low incidence of significant prediction outliers. This suggests that multiview fusion achieves high accuracy and ensures high reliability for legal age thresholds. In contrast, the elevated RMSE observed in the senior group (53–60 years) quantitatively reflected the increased biological variability and the impact of pulp calcification, which introduced non-linear noise that even advanced fusion architectures find challenging to fully address. These metrics, coupled with a global $R^{2}$ of 0.641, confirm that our model effectively captures the variance in secondary dentin deposition across the adult lifespan.

The residual distribution (Fig. 6) reflects the “attraction to the mean” phenomenon, or the Leonets effect, common in forensic regression. However, the lower dispersion of our model ($SD = 3.66$ years in the 23–27 group) indicates a more stable prediction than previous Random Forest approaches. From a forensic standpoint, the tendency to overestimate the age of young individuals must be balanced against the model’s high precision. The Bland-Altman analysis (Fig. 4) serves as a calibration tool, defining “safe zones” where the predictive error is minimized, thus mitigating the risk of “false adult” classifications in criminal contexts.

A key differentiator of this study is the reporting of the standard deviation (SD) as a measure of population-based predictive uncertainty ($5.49 \pm 4.32$ years). While the baseline study [18] reported fold-wise variability (approximately $\pm 0.15$), our choice to report the SD of individual residuals provides a transparent measure of reliability for individual forensic cases. This aligns with international standards for forensic evidence admissibility, as it quantifies the expected margin of error for a specific subject, rather than just the stability of the training algorithm.

The observed “performance crossover” at age 33 reveals a compelling trade-off between model complexity and signal-to-noise ratio. While mult-view fusion is essential for capturing the complex geometries of wide pulp chambers in the young, the single-view model proved more resilient in senior cohorts (48–60 years). In the 53–60 group, the single-view model achieved a MAE of 8.90 years, outperforming both the baseline (9.54 years) and the multi-view model (10.18 years). This suggests that as the pulp cavity undergoes physiological obliteration, the integration of multiple views may introduce morphological noise from the constricted root canals. In contrast, the single-planar representation seems better suited to extract subtle textural features of mineralized dentin, which become the primary age indicators in older individuals [2].

The interpretability provided by Grad-CAM (Fig. 5) confirms the biological validity of the framework. The focus on the cervical region and pulp chamber floor aligns with established odontological knowledge regarding the sites of the most active secondary dentinogenesis. Despite the challenges in geriatric cases due to pulp calcification and restorations, the proposed method establishes a new state-of-the-art for the IPCTI dataset, with a global MAE reduction of 6.8%. Future efforts should focus on hybrid attention mechanisms to further refine the detection of mineralized structures in the senior population, bridging the remaining gaps in geriatric age estimation.

A limitation of this study is the uneven cohort distribution in the IPCTI, leading to increased variance and reduced statistical confidence in underrepresented age groups, particularly older adults. Although the benchmark results were encouraging, this study did not establish a forensic casework reference standard. This method requires external validation, assessment under different conditions, and clear uncertainty reporting before operational use.

## Conclusion

This study presents an innovative multimodal deep fusion method for automated DAE using CBCT imaging. By employing a dual-stream EfficientNet-V2 architecture to synchronize the coronal and sagittal planes, we enhanced the model’s ability to capture the nonlinear patterns of secondary dentin deposition. Integrating clinical metadata and the automated PTR index yielded a peak precision of 4.12 years for the 23–27 age cohort and a 31.3% reduction in MAE for the 18–22 demographic compared to state-of-the-art baselines. Furthermore, the interpretability of Grad-CAM confirmed that the framework prioritizes biologically relevant markers, such as the cervical region and pulp chamber floor, ensuring an objective and reproducible diagnostic process.

Our findings indicate a specialized performance profile: the multi-view approach excels in young adults (18–32 years) by reducing the absolute error by over 2.2 years, while the single-view model offers superior stability for geriatric cohorts, where pulp obliteration introduces noise. Consequently, we recommend a stratified forensic application: the use of the multi-view specialist model for high-precision legal thresholds in young adults and the single-view generalist model for broader identification. Although challenges remain in elderly cases, this approach bridges the gap between deep learning and forensic analysis. Future research should incorporate diverse ethnic metadata and transformer-based architectures to further refine the accuracy across the adult lifespan.

## Footnotes

1 doi.org/10.17632/bjxgd4nyfg.2
